# Increasing Integrative Negotiation in European Organizations Through Trustworthiness and Trust

**DOI:** 10.3389/fpsyg.2021.655448

**Published:** 2021-06-23

**Authors:** Patricia Elgoibar, Francisco J. Medina, Martin Claes Euwema, Lourdes Munduate

**Affiliations:** ^1^Department of Business, University of Barcelona, Barcelona, Spain; ^2^Department of Social Psychology, University of Seville, Seville, Spain; ^3^KU Leuven, Leuven, Belgium

**Keywords:** integrative negotiation, trust, trustworthiness, employee representatives, negotiation outcomes

## Abstract

Integrative negotiation in which employers and employees create value is a major necessity in the current challenging context. Collective labor negotiations in organizations are traditionally focused on mostly distributive issues, such as pay, working hours, and holidays. However, the current situation demands the inclusion of other issues of a potentially more integrative nature, such as telework, sustainability, and risk prevention, the enhancement of which is a major challenge for organizations. In this study, we explore the negotiation process between management and employee representatives (ERs), analyzing the roles of trust and trustworthiness. We collected data from 614 human resources managers from different organizations in 11 European countries. The results confirm that ERs who management perceive to be trustworthy have a greater influence on negotiation, particularly with regard to integrative as opposed to distributive issues, and that trust partially mediates this relationship.

“*Building confidence through trust and dialogue is crucial to making policy measures effective. A human-centered recovery (from the COVID-19 pandemic) is best guaranteed when those affected – through their representatives – have a seat at the table” (ILO*, *2020**, p. 10)*

## Introduction

We are facing unprecedented circumstances due to the COVID-19 pandemic. This is undoubtedly affecting the way organizations work and putting pressure on the working conditions of the near future (ILO, 2020), and the way social agents negotiate these new conditions is becoming even more decisive than ever. The agendas of European collective negotiations have traditionally been centered on classic topics such as working hours, wages, holidays, and performance (Cruz Villalón, 2019). However, integrative issues such as teleworking, training, and flexible working hours are currently being included on these agendas too. These issues have greater integrative potential, and thus generate opportunities to create value in organizational negotiations, where both parties gain (Brett, 2014; ILO, 2020). At the organizational level, these issues are put on the negotiation agenda by both management, typically HR, and employee representatives, hereinafter ERs (Conchon, 2011). This is the level at which we frame the present study. Our first goal is to understand how the parties, particularly ERs, can boost their influence, especially on issues with integrative potential in collective negotiation at the organizational level.

Secondly research suggests that integrative bargaining happens when parties share information and understand priorities—what issues are more or less important for the other party—and use that information to generate trade-offs that create value (Brett, 2014). This is a key factor for achieving integrative agreements in negotiation, which requires trust between parties (Hempel et al., 2009; Lewicki and Polin, 2013) and that at the organizational level is understood to mean “the willingness to risk increasing his or her vulnerability to others whose behavior is beyond one's control. Thus, parties are confident that the other will not exploit the party's vulnerabilities” (Lewicki et al., 2016, p. 96). Therefore, in collective negotiation at the organizational level, where both parties negotiate different distributive and integrative issues, management's trust in ER's is based on its confidence that its vulnerabilities, such as vital information, or dependency in crisis circumstances, will not be exploited by the ERs.

Trustworthiness has been defined as “the characteristics and actions of the trustee [that] will lead that person to be more or less trusted” (Mayer et al., 1995, p. 717). Research on trustworthiness suggests that people judge others based on three dimensions: their ability (competence), benevolence (respectful treatment), and integrity (honesty, consistency of words and actions, willingness to keep promises, etc.) (Mayer et al., 1995; Lewicki et al., 2016). The literature further suggests that perceptions of another party's trustworthiness are central for understanding the emergence of trust (Colquitt et al., 2007), as these perceptions promote positive expectations that the other party will act in honest and non-exploitative ways (Fulmer and Gelfand, 2012; De Cremer et al., 2018). The second goal of the present study is to analyze these specific relationships between trust and trustworthiness in collective negotiation. To the best of our knowledge, this is the first study to analyze this relationship in the field of collective negotiation, addressing, first, the social relevance of the topic and second, the advancement of knowledge in this field. Indeed, the building of confidence through trust and dialogue to explore integrative agreements in collective negotiation is a requirement when dealing with the unprecedented circumstances arising from the COVID-19 pandemic (ILO, 2020). At the same time, various researchers have underpinned the need to analyze the development of intergroup trust in the context of organizational settings (Fulmer and Gelfand, 2012; Guest, 2016).

To conduct this study, we analyze data from HR managers in 11 European countries. The main objective is to explore collective negotiation processes between management and ERs in European organizations, analyzing the role of trust and trustworthiness as antecedents of ER influence on distributive and integrative issues. In the following section, we review the theoretical framework, explaining the concepts of distributive and integrative negotiation, and the role of ERs at the negotiation table, and draw on social exchange theory (Blau, 1964) to analyze the relationship between trust and trustworthiness as explanatory antecedents of their influence at the negotiation table. The theoretical and substantive arguments of the proposed hypothesis are included in this section.

### Negotiating Distributive and Integrative Issues

Starting from a negotiation theory approach, the classic study by Walton and McKersie (1965) identified two different sub-processes in collective negotiation: distributive and integrative. The distributive aspect refers to how negotiators resolve differences when their interests or positions are in conflict. From the game theory approach (Nash, 1950), this is called the “zero-sum game,” or the “win-lose process” (Fisher et al., 1991), because it involves dividing limited resources—as in *a fixed pie*. The integrative process involves the creation and discovery of joint gains. Parties identify common interests and make trade-offs on differentially valued issues (Neale and Bazerman, 1983). By exchanging information, negotiators can develop accurate judgments about the other party's interests and create mutually beneficial integrative agreements—*expanding the pie* (Thompson et al., 1996). This process is called the “variable-sum game” or “win-win agreement.”

Labor negotiations have traditionally covered the “bread and butter” or sought distributive agreements related to limited resources, such as working hours or compensation and benefits. In making distributive deals, parties assume a fixed pie or fixed amount of resources and negotiate ways to split them—or claim value (Brett, 2014). Walton and McKersie (1965) noted that this process is “usually regarded as the dominant activity in a union-management relationship” (p. 11) and that it refers to dividing limited resources and reconciling opposing objectives (Cutcher-Gershenfeld and Kochan, 2015). Although the division of resources is an important part of negotiation, distribution is only one aspect of the process. Integrative negotiation occurs when negotiators *expand the pie—or create value* in negotiations (Brett, 2014). They typically do so either by breaking a single issue into multiple issues or by adding new ones. In either case, when one issue is more important to one party (e.g., keeping jobs in the case of ERs) and another is more important to the other (e.g., maintaining productivity by teleworking in the case of management), the parties can negotiate a trade-off that meets both of their goals and creates value in negotiation. Purely distributive or purely integrative negotiations are rare; they usually involve a mix of issues that combine both elements (Cutcher-Gershenfeld and Kochan, 2015).

The current circumstances have presented a number of creative ways to reach integrative agreements. One is the possibility of recovering working hours lost during the toughest phase of the pandemic in exchange for keeping jobs in the organization (ILO, 2020). Another is investment in online training during periods of the pandemic when economic activity was low. In these cases, the parties refocused the negotiation from the single issue of how many hours could be recovered—a distributive issue as it is related with the parties' share of the available resources—by identifying the multiple issues regarding training or teleworking during the pandemic that turned out to be the real issues in these negotiations—issues with integrative potential as they are able to create added value and maximize joint gains. In sum, distributive issues are those where the earnings of one party are inversely linked with losses by the other, such as working hours, pay, incentive systems, or performance targets. In contrast, issues with integrative potential are those where the parties' interests could differ—and they negotiate a trade-off that creates value in negotiation—, for example work-life balance, equality, and corporate social responsibility (Fisher et al., 1991).

Negotiation studies show that although negotiation in organizations involves distribution of some resources, there are many opportunities for integrative negotiations if negotiators are able to transform single issues into multiple issues and make trade-offs (Brett, 2014). We observe this trend in collective negotiation, where there is greater social awareness within organizations (Martínez Lucio, 2016) of such issues as gender equality (Williamson and Baird, 2014), health and safety, and employee training (Heyes, 2013).

### Influence of Employee Representatives on Collective Negotiation

Employee Representatives are one of the main stakeholders in organizational negotiation, particularly in Europe, and are legally endorsed parties in this context. The employees of any organization in the European Union with more than 50 employees have the right to elect their representatives to the so-called works council (Directive 2002/14/EC), which meets with top management to discuss relevant topics that affect the employees as well as the organization as a whole. In most countries, ERs are elected for a period of 4 years to represent their co-workers in different types of organizational conflicts, negotiations, and decision-making processes (Conchon, 2011). Their role has become more complex and diverse, and is often a response to greater social awareness within organizations (Martínez Lucio, 2016; Nauta et al., 2016). Typically, framework negotiations take place at the sector level between unions and sectors in a given industry. However, more specific arrangements are negotiated at the organizational level, where the works council plays a crucial role.

In this organizational context, ERs and management are long-term partners. Somebody might leave their position at the negotiation table, but the negotiations continue with someone else representing their group (Lewicki et al., 2016). Secondly, in terms of the control of resources, this relationship is characterized by power imbalance. In relationships between management and ERs, the latter are commonly more vulnerable to the actions of the former due to differences in power, status, and control of resources (Lapidot et al., 2007). From this hierarchical perspective, management has the more dominant position because it has more information about the company's strategy, financial reports, or future expectations (Fells and Prowse, 2016). Indeed, information sharing has been positively related to higher quality of discussions and decision making (Moye and Langfred, 2004), and lack of information sharing is directly related to a lack of trust between the parties (Munduate et al., 2012). However, ERs represent their coworkers and are legally entitled to a seat at the negotiation table, which fosters their influence on collective negotiation and tends to balance the role played by the two parties in the negotiation process (Munduate and Medina, 2017). Finally, ERs and management often have different traditions and values with regard to negotiation. Workers view it as a protective element, and a means to have a say in the organization's decision-making processes, while managers might perceive it as a way to gain organizational support and overcome resistance. Managers who do not see the benefit of including workers in decision-making processes may view consultations and negotiations as time-consuming and restricting of their power and flexibility, and as a legal obligation. Therefore, given the characteristics of collective negotiation in the context of employment relations, trust is an important feature of the process (Guest, 2016). Trust between parties makes progress toward an agreement more likely, attains more progressive agreements, and increases the prospects of mutually positive outcomes (Hempel et al., 2009; Kong et al., 2017). Based on social exchange theory and the principle of reciprocity, we elaborate on how trust and trustworthiness are key mechanisms that could explain the influence of ERs on negotiation of both distributive and integrative issues.

### Intergroup Trust, Trustworthiness, and Reciprocity in Collective Negotiation

As Fulmer and Gelfand (2012) point out, the distinction between trust at the individual level and trust at the group level is critical. For example, Song (2009) found that teams have less trust in one another than individuals do. They also highlighted the distinction between teams as a referent and as a level of analysis. As a referent, trust in teams can be measured as trust in an individual, in the team in general or at the level of the entire organization. As a level of analysis, trust at team level refers to trust that is shared between all the members of the team, regardless of the referent (Fulmer and Gelfand, 2012). In collective negotiation, where ERs and management act as representatives of their respective groups, the team is considered to be the referent—employees on the one hand and management on the other—and intergroup trust is analyzed at the individual level, because the collective negotiation is performed at an interpersonal level, between representatives of the management and of the employees.

Compared with interpersonal trust, intergroup trust has only received scarce research attention in organizational settings (Kramer, 1999; Dirks and Ferrin, 2002; Fulmer and Gelfand, 2012). While it is important to examine individuals, we explore whether trust in a group or team and team trustworthiness can influence the outcomes of collective negotiations, also in an organizational setting. Indeed, some authors stress the importance of extending trust to the group and the organization as referents, and as levels of analysis, and call for additional research (Guest, 2016). We understand that some theoretical and contextual characteristics of these intergroup perspectives are important for collective negotiation outcomes. First, social exchange theory and the principle of reciprocity (Blau, 1964) are recognized as an analytic framework for the analysis of intergroup trust (Guest, 2016; Lu et al., 2017). Second, we echo the message with respect to the importance of examining the development of intergroup trust in the organizational setting. As Fulmer and Gelfand (2012) noted, “the antecedent of trust in teams….may depend on the context” (p. 1189). Colquitt et al. (2011) for example, found that the consequences of team members' integrity and benevolence for trust in teams depend on the unpredictability and risk of the task context. Likewise, Lapidot et al. (2007) found that the relative importance of integrity and ability for building trust depends on the situational trustor's vulnerability.

Based on social exchange theory, as first outlined by Blau (1964), “individuals trust another entity, based on what they put into and what they receive from a relationship” (Fulmer and Gelfand, 2012, p. 1175). A central theme in this theory is that employees and employers may develop exchanges for social or economic reasons. Influence on negotiation outcomes can be viewed as socially motivated, especially with regard to integrative outcomes because they create value in negotiation (Munduate et al., 2016). However, social exchange leads to risk-taking by the trustor and there is a need for trust in order for it to operate to the benefit of both parties. Gouldner (1960) argued that over time the social exchange process becomes influenced by “the norm of reciprocity” whereby each party feels obliged to reciprocate positive acts by the other party, thereby reinforcing levels of trust (Guest, 2016). We can conclude that trust is built through a process of reciprocal exchange and mutual influence that negotiators have toward one another (Korsgaard et al., 2015; Lu et al., 2017). Building on the definitions of trust (Lewicki et al., 2016) and trustworthiness (Mayer et al., 1995) identified earlier, we suggest that management's trust reflects the extent to which it perceives the ERs to be trustworthy, and how much it feels that their ability, benevolence, and integrity make them a favorable exchange partner (Mayer et al., 1995).

These characteristics have been defined as the core components of trustworthiness. Ability concerns “the group of skills, competencies, and characteristics that enable a party to have influence within some specific domain” (Mayer et al., 1995, p. 717). It entails functional (e.g., task-specific) as well as interpersonal competencies (e.g., people skills). Benevolence is defined as “the extent to which a trustee is believed to want to do good to the trustor, aside from an egocentric profit motive” (Mayer et al., 1995, p. 718). This suggests a fundamental aspect of an interpersonal or intergroup relationship (Levin et al., 2006; Knoll and Gill, 2011), in which the trustee has a specific attachment to the trustor (Mayer and Davis, 1999), implicating expectations of benevolent motives on the part of the other (Rousseau et al., 1998; Yamagishi, 2011; Balliet and Van Lange, 2013). Furthermore, integrity concerns the trustor's belief that the trustee is dedicated to an acceptable set of moral and ethical principles (Colquitt et al., 2007; Mayer et al., 2011). It represents a rational trust of the other party, because the feeling of fairness or morality can entail a long-term predictability that can help individuals cope with the uncertainty of trusting another party (Lind, 2001). These three components of trustworthiness are positively related to trust (Mayer et al., 1995; Knoll and Gill, 2011; Fulmer and Gelfand, 2012). We argue that in collective negotiation, the perception of abilities, benevolence, and integrity by management will promote positive expectations that ERs will act in honest and non-exploitative ways, and managers will therefore be more inclined to take the risk of trusting them. Following social exchange theory and the norm of reciprocity (Blau, 1964) the manager will then allow the ERs to have a greater influence on collective negotiation outcomes. Although the relationship between trustworthiness and trust has been extensively studied (see the meta-analysis by Colquitt et al., 2007), it has not been applied to a collective context in organizations. Thus, the first step is to confirm that trustworthiness does indeed also act an antecedent when managers are the trustors and the ERs are the trustees.

*Hypothesis 1: Trustworthiness is positively related to trust in collective negotiation*.

The ability, benevolence, and integrity of ERs, as a team, have a critical impact on the extent to which managers allow them to influence negotiation outcomes. The literature suggests that the ability to create an environment of trust leads to integrative potential in negotiation (Pruitt and Carnevale, 1993; Tinsley et al., 2006; Welsh, 2012). Welsh (2012), for example, concludes that “a negotiator's perceived trustworthiness (…) is likely to be positively correlated with his or her effectiveness as an integrative negotiator” (p. 136). The influence of ERs on negotiation outcomes and especially on integrative outcomes is linked to the conceptualization of negotiation as a social exchange process. Building on the social exchange theory (Blau, 1964) identified earlier, we have seen that employees and employers may develop exchanges for social or economic reasons. Traditionally, exchange is perceived in terms of economic value. That is, economic outcomes that address financial needs are typically contractual and tend to be tangible, such as wages or working conditions. However, exchanges can stand for more than plain material needs (e.g., organizational investment in one's career), and might also address the parties' social needs. For example, Organ and Konovsky (1989) state that organizational fairness fosters a sense of trust on the part of the employees, involving a mutual provision of diffuse, vaguely defined obligations that are delivered over an open-ended time frame. Social outcomes also send the message that the other party is appreciated and/or treated with dignity (Cropanzano and Mitchell, 2005; Shore et al., 2006). According to Blau (1964), social exchanges entail unspecified obligations whereby when one partner does another party a favor, something is expected in return. For example, following Organ and Konovsky (1989), if ERs are treated fairly, then they should be expected to respond reciprocally. Similarly, if ERs are perceived to be trustworthy—in terms of their ability, benevolence, and integrity—then they should expect to be allowed to influence negotiation outcomes. The social exchange literature in several fields (Meyer and Allen, 1997; Rousseau et al., 1998; Guest, 2004, 2016) does not consider the inclusion of the social dimension to imply the exclusion of the economic dimension, but rather that the two may be operating concurrently. Both aspects of exchange are core to the new social contract in employment relations and are gaining relevance in today's workplace (Arenas et al., 2017). Therefore, organizations are now engaged in negotiating new issues (ILO, 2020) and innovative processes (Rubinstein and McCarthy, 2016) as part of the social dimension exchange. In fact, for distributive issues at the organizational level, in most European countries the existing legislation makes the position of ERs and the works council clear (Cruz Villalón, 2019). Trustworthiness might therefore be less relevant for influencing these issues, as it is established by regulations. However, integrative issues in which parties can influence and create value have become an important part of the current circumstances. During the pandemic, teleworking, vocational training, and work-family balance are relevant and can have integrative potential. Here, the influence of ERs is key and flexible, because depending on the influence granted, they can participate and create value in the negotiation processes. We therefore expect ER trustworthiness to be more strongly related to integrative, rather than distributive issues.

*Hypothesis 2: Trustworthiness has a stronger relation to the influence of ERs in collective negotiations on integrative issues than on distributive issues*.

Interpersonal trust in the workplace has been shown to have a strong and robust influence on a variety of organizational phenomena including decision-making effectiveness (Alge et al., 2003), constructive conflict resolution (Tsai and Ghoshal, 1998; Euwema et al., 2015), and information sharing (Howorth et al., 2004; Zaheer and Zaheer, 2006), among others. Trust is viewed as one of the most influencing variables in employment relations (Walton et al., 1994; Dirks and Ferrin, 2002; Guest, 2004; Ferrin et al., 2007; Hempel et al., 2009; Fulmer and Gelfand, 2012). Some studies have dealt with trust as a dependent variable in the realm of employment relations. For example, Laplante and Harrisson (2008) examined how trust between managers and union representatives is built, and Guest et al. (2008) examined the relationship between partnership practices and labor-management trust. Knoll and Gill (2011) studied the importance of trustworthiness in predicting trust in supervisors, subordinates, and peers. Hence, the ability to develop trust has become a critical competence in employment relations (Lewicki et al., 1998; Elgoibar et al., 2016).

Through trust, parties can be confident to be open with each other, because they know that the information they have shared will not be used against them (Zaheer and Zaheer, 2006). The strategy of constructive controversy highlights the advantages of open-minded discussions, listening carefully to others' opinions, and trying to understand their views (Tjosvold et al., 2014). Trust also induces members to rely on each other's commitments (Gibson and Birkinshaw, 2004). Trusting and collaborative relations between ERs and management are also critical to improve performance outcomes in organizations (Rubinstein and McCarthy, 2016). Previous studies in the area conclude that trust should be addressed explicitly while intervening to prevent conflicts or the escalation thereof and instead searching for constructive agreements (Nauta et al., 2016).

In the context of collective negotiation, there is a need to understand the underlying mechanism between ERs' trustworthiness and their influence on distributive and integrative issues. Our third hypothesis is based on the two previous ones. On the one hand, following the Mayer et al. (1995) model, trustworthiness is an antecedent of trust as explained by H1, and trust in turn will partly explain the ERs' influence on negotiations. On the other hand, in H2 we explore the direct relation between trustworthiness and influence on distributive and integrative issues. By combining these two previous arguments, we expect trust to play a partial mediation role in the relation between trustworthiness and influence in distributive and integrative issues. That is, trustworthiness will indeed directly impact the influence of ERs and at the same time trust will partially explain the relationship with both outcomes: influence on distributive and integrative issues. Hence, we propose the following hypothesis.

*Hypothesis 3. Trust plays a partial mediation effect in the relationship between trustworthiness and influence of ERs on (a) distributive and (b) integrative issues*

## Method

### Procedure and Sample

In order to test our hypotheses, data were collected through an online survey in 11 European countries: Belgium (*N* = 65), Denmark (*N* = 103), Estonia (*N* = 52), France (*N* = 40), Germany (*N* = 33), Italy (*N* = 42), the Netherlands (*N* = 70), Poland (*N* = 58), Portugal (*N* = 45), Spain (*N* = 84), and the United Kingdom (*N* = 22). In all countries, HR directors and managers from different sectors and of different sizes were invited to participate using different networks in each participating country. We followed random sampling procedures in each country. We contacted employer associations and sent individual invitations to participate in the survey via their personal emails. We focused on CEOs and HR managers as they deal most frequently with ERs in most organizations and are engaged in most negotiations. Overall, 614 HR managers completed the survey. The average age of participants was 43.5 years, and they were 50% male and 47% female (3% did not answer). The survey and instructions were translated into 10 languages, through back-and-forth translation by native experts (Danish, Dutch, English, Estonian, French, German, Italian, Polish, Portuguese, and Spanish). For Belgium, both Dutch and French surveys were made available. In addition to measuring the key variables, information on participants (age, gender, role, education, years actively in contact with ERs), and organizations (number of employees, economic conditions) was gathered.

### Measures

#### Trustworthiness

We selected two items of each dimension of the trustworthiness scale developed by Mayer and Davis (1999). The selection was based on theoretical and empirical reasons, selecting the most representative item of each dimension. The items measuring the abilities of ERs were: “Employee representatives are capable of performing their job as representatives” and “Employee representatives are well-qualified to perform their role as representatives.” The items measuring benevolence of ERs were: “Employee representatives look out for what is important to the organization” and “Employee representatives would not do anything (deliberately) to hurt the organization.” Items measuring the integrity of ERs were: “Employee representatives have a sense of justice” and “Employee representatives will stick to their word.” Items were scored on a 5-point scale (1 = Totally disagree, 5 = Totally agree).

#### Trust

Trust was assessed with a 3-item scale adapted from the Organizational Trust Inventory (Nyhan and Marlowe, 1997). A sample item is: “To what extent is there a trusting relationship between management and employee representatives?” Items were scored on a 5-point scale (1 = None, 5 = A very great deal).

#### Influence of Employee Representatives on Distributive and Integrative Issues

To measure the influence of ERs we developed a scale that asked the participants to state the degree of influence of ERs regarding eight issues. Taking into consideration how national systems vary in terms of the respective roles of collective bargaining and legislation regulating the labor market (Eurofound: Welz et al., 2020), for the purposes of the research we selected the topics that Eurofound considers relevant to current industrial relations in Europe (Eurofound: Welz et al., 2020). Given that all countries in the sample belonged to the European Union at the time of data collection, this source accurately represents the topics that ERs can influence. The question was: “*To what extent do employee representatives have an impact in your organization on the following subjects?”* As mentioned before, organizational issues embody a wide area of possible discussion points. The items in the questionnaire were measured on a Likert scale from 1 to 5 (1 = No impact, 5 = Strong impact). Three items covered influence on distributive issues: working hours, pay and incentives, and performance targets. A further five items were included to investigate integrative issues: training and career development, health and safety, work-life balance, equality issues, and corporate social responsibility.

## Results

Before analyzing the hypotheses, we conducted an exploratory factor analysis following Field (2013). A principal axis factor analysis was conducted on the 17 items. The Kaiser–Meyer–Olkin measure verified the sampling adequacy for the analysis, KMO = 0.88, which is well above the acceptable limit of 0.5 (Field, 2013). An initial analysis was run to obtain eigenvalues for each factor in the data. Four factors had eigenvalues over Kaiser's criterion of 1 and in combination explained 65.3% of the variance. We retained four factors because of the large sample size and the Kaiser's criterion on this value. Table 1 shows the factor loading after rotation. The items that cluster on the same factor suggest that factor 1 represents trustworthiness, factor 2 represents impact on integrative issues, factor 3 represents trust, and factor 4 represents impact on distributive issues. Given the low loading of the item: “*To what extent do employee representatives and management distrust each other?”* we decided to delete it from the analysis. We also conducted a reliability analysis of the scales, which revealed that trustworthiness has good reliability (Cronbach's α = 0.87); trust has acceptable reliability (Cronbach's α = 0.76) although when deleting the item suggested by the factor analysis (“*To what extent do employee representatives and management distrust each other?”)*, reliability improves to good (Cronbach's α = 0.88); influence on distributive issues has acceptable reliability (Cronbach's α = 0.74); and finally, influence on integrative issues has good reliability (Cronbach's α = 0.86).

**Table 1:** Rotation factor matrix.

**Item**	**1**	**2**	**3**	**4**
To what extent is there a trusting relationship between management and employee representatives?	0.390	0.128	**0.817**	
To what extent is there constructive dialogue between management and employee representatives?			**0.720**	
To what extent do employee representatives and management distrust each other?			0.387	−0.107
Employee representatives are capable of performing their jobs as representatives.	**0.624**		0.304	
Employee representatives are well-qualified to perform their roles as representatives.	**0.666**			
Employee representatives look out for what is important to the organization.	**0.751**			
Employee representatives would not do anything (deliberately) to hurt the organization.	**0.668**	0.103	0.139	
Employee representatives have a sense of justice.	**0.748**	0.148	0.149	
Employee representatives will stick to their word.	**0.669**	0.117	0.221	
To what extent do employee representatives have an impact in your organization regarding: Working hours?		0.377		**0.532**
To what extent do employee representatives have an impact in your organization regarding: Pay and incentives?		0.206		**0.808**
To what extent do employee representatives have an impact in your organization regarding: Performance targets?	0.136	0.404		**0.538**
To what extent do employee representatives have an impact in your organization regarding: Training and career development?	0.103	**0.609**		0.330
To what extent do employee representatives have an impact in your organization regarding: Health and safety?	0.109	**0.651**	0.101	0.197
To what extent do employee representatives have an impact in your organization regarding: Work-Life balance?	0.169	**0.741**		0.237
To what extent do employee representatives have an impact in your organization regarding: Equality Issues?	0.165	**0.846**		
To what extent do employee representatives have an impact in your organization regarding: Corporate Social Responsibility?	0.304	**0.605**	0.158	0.143

*Extraction method: principal axis factoring; Rotation method: Varimax with Kaiser Normalization. Bold values indicate the highest factor loading for the item*.

To analyze the hypotheses, we used the following procedures. Hypothesis 1 was tested using Pearson correlation analysis. Hypothesis 2 was tested using Pearson correlation analysis and Cohen's test to check the significance difference between the correlations. Hypothesis 3 was tested using Hayes (2012) PROCESS macro, which allows us to compute the proportion of variance explained by the model for influence on both distributive and integrative issues, as well as to explore the significance of the indirect effect. We first test the potential meditation effect of trust in the relationship between trustworthiness and influence on distributive issues. We then did the same for the relationship between trustworthiness and influence on integrative issues. We explain the results of these procedures below.

Table 2 presents the descriptive statistics as well as the correlations of the examined variables. Regarding H1, the participants scored *M* = 3.32, *SD* = 0.79 for trustworthiness and *M* = 3.31, *SD* = 0.93 for trust. Bias corrected and accelerated bootstrap 95% CIs are reported in square brackets. Trustworthiness was significantly correlated with trust *r* = 0.61, 95% BCa CI [0.55, 0.67], *p* <0.001. H1 is confirmed.

**Table 2:** Descriptives and correlation analysis between examined variables (*N* = 614).

	**Mean**	**Sd**	**Trustw**.	**Trust**	**Distributive**	**Integrative**
Trustw.	3.32	0.79	/	0.615^*^	0.143^**^	0.386^**^
Trust	3.31	0.93		/	0.150^**^	0.335^*^
Distributive	2.63	0.89			/	0.549^**^
Integrative	2.88	0.84				/

** *p < 0.01*,

* *p < 0.05*.

Regarding H2, the participants scored relatively low for the dependent variable influence of ERs: on distributive issues with a score of *M* = 2.63, *SD* = 0.89; and on integrative issues *M* = 2.88, *SD* = 0.84. Bias corrected and accelerated bootstrap 95% CIs are reported in square brackets. Trustworthiness was significantly correlated with influence on distributive issues *r* = 0.14, 95% BCa CI [0.05, 0.23], *p* < 0.001 as well as with influence on integrative issues *r* = 0.39, 95% BCa CI [0.28, 0.47], *p* < 0.001. To confirm the difference in significance of the correlation between trustworthiness and both distributive and integrative issues, we conducted Cohen's analysis (Cohen, 1988). Cohen suggests an effect size measure with the denomination *q* that can be used to interpret the difference between two correlations. The two correlations are transformed with Fisher's *Z* and subtracted afterwards. We first analyzed Fisher's *Z* and the result was *Z* −4.59; *p* < 0.001. Next, we calculated the effect size, and the results of Cohen's *q* was *q* = 0.263. The interpretation of this result allows us to confirm that the difference in correlations is small but significant. Our data supports the notion that trustworthiness has a stronger impact on the influence on integrative rather than on distributive issues. H2 is confirmed.

**Figure 1:**
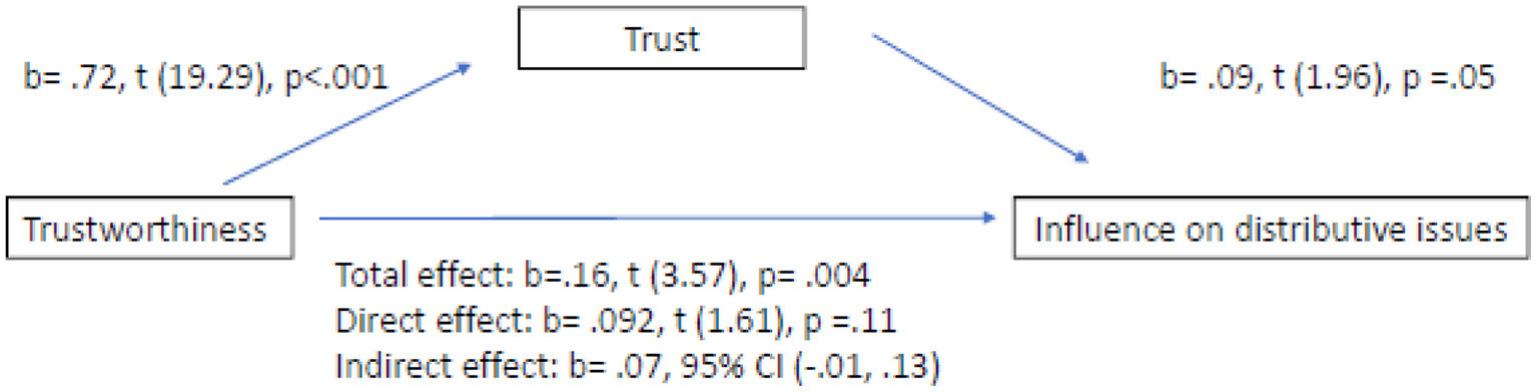
Total effect vs. direct effect vs. indirect effect on influence on distributive issues.

Regarding H3a, the bootstrapping analysis did not present any evidence for the mediation effect of trust in the relationship between trustworthiness and distributive issues. In Step 1 of the mediation model, the regression of trustworthiness regarding influence on distributive issues, ignoring the mediator (total effect), was significant, *b* = 0.16, *p* = 0.004 and the R-sq indicated that trustworthiness explains 2% of the variance of influence on distributive issues. Step 2 showed that the regression of trustworthiness regarding the mediator (trust) was significant, *b* = 0.72 *p* < 0.001, in line with H1 and the R-sq indicated that trustworthiness explains 37% of the variance of trust. Step 3 of the mediation process showed that the mediator (trust), controlling for trustworthiness, was non-significant, *b* = 0.09, *p* = 0.05. Step 4 of the analysis revealed that, controlling for the mediator (trust), trustworthiness was not a significant predictor of influence on distributive issues *b* = 0.09, *p* = 0.11. Indirect effect is not significant, *b* = 0.07, CI (−0.01, 0.13). A Sobel test was conducted and found no mediation effect in the model (*z* = −0.05, *p* = 0.96). It was found that trust does not mediate in the relationship between trustworthiness and influence on distributive issues. H3a is not confirmed (Figure 1).

**Figure 2:**
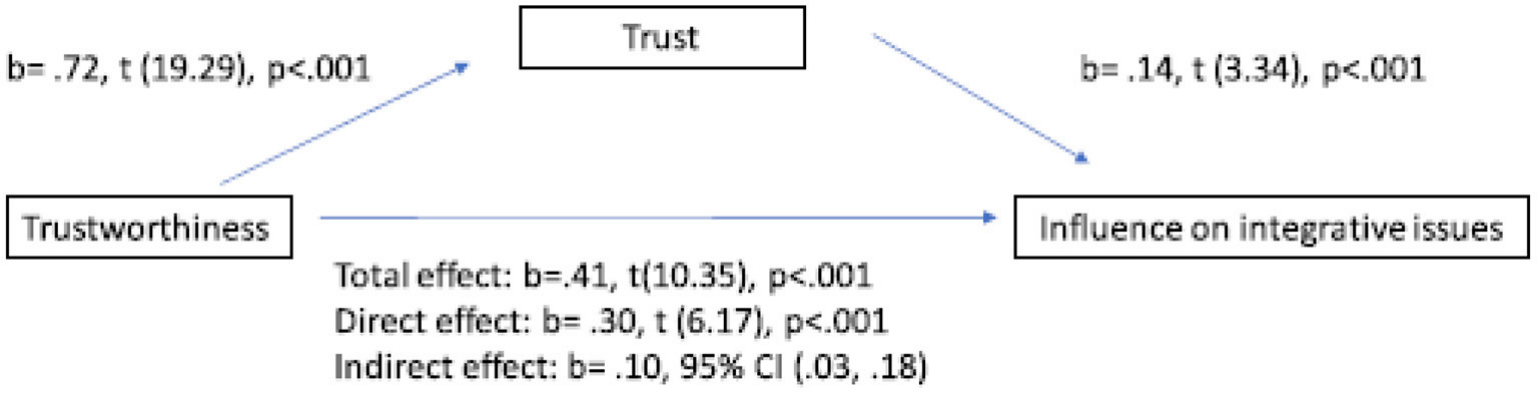
Total effect vs. direct effect vs. indirect effect on influence on integrative issues.

Regarding H3b, the bootstrapping analysis found a partial mediation effect of trust in the relationship between trustworthiness and integrative issues. In Step 1 of the mediation model, the regression of trustworthiness regarding influence on integrative issues, ignoring the mediator (total effect), was significant, *b* = 0.41, *p* < 0.001, and the R-sq indicated that trustworthiness explains 14% of the variance of influence on integrative issues. Step 2 showed that the regression of trustworthiness on the mediator (trust) was significant, *b* = 0.72, *p* < 0.001, in line with H1, and the R-sq indicated that trustworthiness explains 37% of the variance of trust. Step 3 of the mediation process showed that the mediator (trust), controlling for trustworthiness, was significant, *b* = 0.14, *p* < 0.001, and the model explains 16% of the variance of influence on integrative issues. Step 4 of the analysis revealed that, controlling for the mediator (trust), trustworthiness was a significant predictor of influence on integrative issues *b* = 0.31, *p* < 0.001. There was a significant indirect effect of trustworthiness regarding influence on integrative issues through trust, *b* = 0.10, CI (0.02, 0.18). A Sobel test was conducted and found a mediation effect in the model (*z* = 3.29, *p* = 0.001). This test indicates that the indirect effect between the predictor and the outcome is significant and we can therefore conclude that trustworthiness significantly affects influence on integrative issues via trust (Hayes, 2012). As the direct and indirect effects are still significant, we can conclude that trust has a partial mediation effect in this relation. H3b is confirmed (Figure 2).

These findings support the hypotheses that both trustworthiness and trust are positively related (H1); trustworthiness is related to the influence on both distributive and integrative issues, this relationship being stronger with regard to integrative issues (H2); and that trust partially explains the impact of trustworthiness on influence on integrative issues (H3b), but not on distributive issues (H3a).

## Discussion

In this study we explored the negotiation process between management and ERs, analyzing the role of trust and trustworthiness in promoting integrative agreements in organizational settings. Our first aim was to understand how the parties, and particularly ERs, can boost their influence, especially regarding issues with integrative potential in collective negotiation at the organizational level. We clearly identified that ERs' trustworthiness impacts the influence granted by management in collective negotiations. This is important, given that this influence on collective negotiations can impact employees' work and lives. What is more clearly novel in these data is that this influence is stronger for integrative issues than distributive issues. This finding is particularly useful considering the current circumstances of global pandemic in which management and ERs need to negotiate issues with integrative potential, such as teleworking in order to maintain jobs and to keep the workers and the general population safer. The theoretical argument behind the positive relationship between ERs' trustworthiness and influence on negotiation outcomes is explained by social exchange and reciprocity theories (Gouldner, 1960; Blau, 1964). As Colquitt et al. (2007, p. 911) suggested “many of the facets of trustworthiness can be viewed as currencies that help create a social exchange. For example, trustworthiness facets such as demonstrating concern and support or acting based on sound principles can be viewed as actions that should engender a motivation to reciprocate on the part of an exchange partner.”

Secondly, we wanted to explore whether there is a positive relationship between trustworthiness and trust in collective negotiations at the organizational level, particularly between ERs' trustworthiness as perceived by management. The answer is a qualified yes. The more ERs as perceived to be trustworthy by management, the greater the latter's trust in them. This positive relationship is not surprising, given prior findings in other contexts such as employees-top management (Mayer and Davis, 1999); leaders and followers (Heyns and Rothmann, 2015); or university students (Jones and Shah, 2016). However, it is useful to confirm that it also occurs in collective negotiations between parties at the organizational level. To our knowledge, there are no previous studies that have explored the relationship between ERs' trustworthiness and management's trust. Given the relevant role of ERs in collective negotiation and the importance of trust in negotiation (Druckman et al., 2019), these results are of interest on a theoretical and practical level. These two constructs are also strongly related in the context of collective negotiations, the conclusion being that the trustworthiness perceived by the trustee impacts the trust granted by the trustor, leading the trustor to accept greater vulnerability and establish a trusting relationship (De Cremer et al., 2018). Management's perceptions of the extent of their ERs' trustworthiness, a characteristic that makes them a favorable exchange partner, are central for understanding the emergence of trust (Colquitt et al., 2007; De Cremer et al., 2018). Following Colquitt et al. (2007) we can also conclude that “the concept of trustworthiness is central to understanding and predicting trust levels” (p. 910) in the context of collective negotiations between parties in organizations.

Finally, we have explored the role of trust in the relationship between trustworthiness and influence on collective negotiation outcomes. Mediation analyses show that trust plays a partial mediation effect in the relationship between trustworthiness and influence on integrative issues, but not on distributive issues. This could be explained by the context of the trust relationship in collective negotiation, and in particular the long-term perspective of this negotiation process. Research suggests that the consequences of team members' trustworthiness and individuals' trust in teams is moderated by the temporal perspective of the relationship between parties (Jones and Shah, 2016). For example, Sharif et al. (2021) found that trust develops and changes with time (mostly in the form of long-term relationship orientation), through the (positive or negative) impact of cognitive and behavioral actions. In the same line, Jones and Shah (2016) analyzed the relative importance of the trustor, the trustee and the trustor–trustee dyad relationship for trust building, and how the influence of each focus changes over time. They found that trustor influence decreases over time while trustee and dyadic influences increase. Taken the time-related treatment of trust in previous research together with the characteristics of collective negotiation, we consider that this direct relationship could be explained by the long-term perspective in the pattern of reciprocity in collective negotiation. In this context, management's perception of ERs' behavior may be critical for increasing the latter's influence on negotiation outcomes. However, as this study does not include a longitudinal design, we cannot confirm this explanation, which is based on the context of the study. It could also be concluded that trustworthiness has a direct impact on the influence granted to ERs and, in line with previous research (Colquitt et al., 2007), our results suggest that trustworthiness is important even aside from its trust-fostering role. However, it is interesting that trust partly explains the influence granted with regard to integrative issues. This could be explained by the fact that for distributive issues the influence is based on contextual variables such as national law, the previous collective agreement, or the long-standing traditions concerning negotiation issues in a given sector or company. Meanwhile, integrative issues are included much more flexibly in negotiation, and depend to a larger extent on the trust developed with the counterparty, in this case the ERs.

### Theoretical Implications

The first theoretical contribution is to the field of trusting relationships. Based on the theoretical framework of the social exchange and reciprocity principles (Gouldner, 1960; Blau, 1964), as well as the requirement in the literature to study the mechanisms of trust reciprocity at the group level (Fulmer and Gelfand, 2012), the study analyzed the relevance of the social context (De Cremer et al., 2018) in which the trusting relationship occurs. In turn, the reciprocity principle at the group level, between the perception of ERs' trustworthiness and the influence granted by management on negotiation outcomes, has been explored.

A second contribution is the consideration of two different types of issue in which ERs have influence in an organizational setting: distributive and integrative issues. This differentiation has barely been considered in previous studies on collective negotiation, with few exceptions (Cutcher-Gershenfeld and Kochan, 2004; García et al., 2017). Indeed, the diversity of issues is important when analyzing trusting relationships at the negotiation table. We have seen that trustworthiness has a greater impact on influence regarding issues with integrative potential than it does for purely distributive issues. This may suggest that the inclusion of distributive issues is decided upon beforehand in most European countries through the corresponding legislation (Cruz Villalón, 2019) whereas issues with integrative potential offer a higher level of variance and flexibility in terms of ERs' influence. In turn, variables such as trustworthiness and trust play an important role in the increase in ERs' influence on such issues, which are particularly relevant in the current situation, in which organizations need to adapt rapidly to the consequences of the COVID-19 pandemic. Integrative issues such as teleworking, training during periods of low activity, and security are key aspects for ensuring safe jobs and even lives (ILO, 2020). Thus, an understanding of the impact of trustworthiness on ERs' influence when negotiating can help organizations to make better and consensus-based decisions.

This study improves our understanding of how trustworthiness affects the influence of ERs on the issues negotiated at the collective bargaining level. Importantly, we also show the partial mediation effect of trust. This study adds to previous research on trust in industrial relations and the essential role of trustworthiness, as well as applying a model analyzed previously in other areas—the antecedent role of trustworthiness—to a collective negotiation context.

### Practical Implications

The study also makes a major contribution to the field of labor relations. It has been supported by solid theoretical frameworks from the field of organizational behavior, in order to promote the inclusion in collective negotiation of issues with integrative potential. The results have shown that managers' perceptions of the trustworthiness of ERs, and the trust between the parties in collective negotiation, promote the inclusion of integrative issues in negotiation outcomes. These findings have potentially important practical implications. Firstly, considering the relevance of being perceived as trustworthy, trade unions should consider the dimensions of ability, benevolence, and integrity when attracting and recruiting new members for the role of ERs, so that ERs will be granted greater influence in collective negotiations, especially in the case of integrative issues. Secondly, in addition to selecting the right people for the role, the training of ERs in the dimensions of trustworthiness seems an effective way to promote the specific abilities and competencies of the role in order for them to gain influence at the negotiation table, this being is a relevant topic in the field of industrial relations (Munduate et al., 2016). However, our study shows that perceived benevolence and integrity also play a key role in both trust and influence, and so these dimensions should also be promoted through training.

### Limitations and Suggestions for Future Research

Throughout this study, we have investigated managers' views. However, the fact that our results rely only on reports from managers may entail a risk of common method variance (Podsakoff et al., 2003). Hence, it is important to bear in mind that these viewpoints only concern one side of the negotiation table. Nevertheless, the three main concepts of the study—trust, trustworthiness, and influence on negotiation outcomes—are perception driven and the assessment of perceptions can thus provide an accurate measure. For example, trustworthiness is based on beliefs and attitudes, which can be accurate predictors of the future actions of the other party, based on what their intentions are (Caldwell and Hansen, 2010). In future research, these findings could be explored in terms of both the perceptions of ERs and managers to offer a comparative view of the situation. We also encourage further research on the role of trustworthiness and how it could affect different organizational outcomes. As seen in this study, the impact of trustworthiness varies depending on the issues at stake. We are also aware that the fact that the study does not include a longitudinal design affects the explanation for the direct relationship between trustworthiness and influence and the partial mediation effect of trust. Thus, we strongly encourage longitudinal studies in order to analyze how a long-term horizon in the relationship, as is the case in collective negotiations, impacts the evolution of trust and trustworthiness and their impact on the influence on negotiation outcomes. Finally, the study reports data from 11 countries with a diversity of industrial relations systems. Future research could account for this diversity in its designs and observe whether the macrolevel of the system can indeed impact the relationships between trustworthiness, trust, and influence on distributive and integrative issues.

To conclude, our results show that the trustworthiness of ERs has a direct impact on their influence on collective negotiations, and that this influence is higher in relation to issues with integrative potential than it is for traditionally distributive issues. Additionally, trust partially explains the impact of trustworthiness on how ERs influence integrative issues, but not distributive ones. The challenge now is to further explore how the trustworthiness of ERs can be boosted to make their influence on these negotiations a constructive and beneficial factor in organizations both now and in the future.

## Data Availability Statement

The datasets presented in this article are not readily available because these contain confidential information of persons and organizations. Requests to access the datasets should be directed to Dr. Elgoibar.

## Author Contributions

PE carried out the data collection, data analysis, literature review, research design, and writing the manuscript. ME, FM, and LM carried out the data collection, research design, supervised the method, made a substantial, direct, and intellectual contribution through writing and supervising all the stages of the research process and reviewing the several drafts of the article. All authors contributed to the article and approved the submitted version.

## Conflict of Interest

The authors declare that the research was conducted in the absence of any commercial or financial relationships that could be construed as a potential conflict of interest.
